# Can students identify AI? – A cross-sectional quantitative study about AI recognition in tablet-based MCQ assessment among fifth-year undergraduate medical students at Saarland University, Germany

**DOI:** 10.1186/s12909-026-10410-8

**Published:** 2026-09-24

**Authors:** Philip Vogt, Nadine Wolf, Sandra Jordan, Sara Volz-Willems, Johannes Jäger, Fabian Dupont

**Affiliations:** 1 Department of Family Medicine, Saarland University, Geb. 80.2 Saarland University Hospital, Homburg (Saar), 66421 Germany; 2 Institute of General Practice and Family Medicine, Medical Faculty, Martin- Luther- University Halle- Wittenberg, Halle (Saale), Germany

**Keywords:** Medical education, Digital assessment, Large language models, AI, MCQ, Key feature question

## Abstract

**Background:**

Beyond technical feasibility of Artificial Intelligence (AI)-generated multiple-choice questions (MCQs), their educational value in assessment remains unclear. This study aims to evaluate whether students can distinguish between AI-MCQs and National Licensing Exam (NLE) questions in an exam setting and if they align with the curriculum.

**Methods:**

In this cross-sectional study 119 year five medical students completed a Family Medicine MCQ tablet-based exam. Participants answered 30 AI-generated and 30 NLE MCQs. AI questions were generated from digital learning materials using ChatGPT-4o and Gemini 1.5 Pro. Experts accepted 82% of the questions, with minor edits to retained items and removing those needing major changes. During the exam, students were asked about the question source and whether each item aligned with the course curriculum. Statistical analyses were obtained using Jamovi 2.3.28.0.

**Results:**

No significant difference in correct attribution of AI or NLE MCQs (t(29.6) = -1.24, *p* = .225; t(29.6) = 1.18, *p* = .246) was observed. No significant correlation was found between item difficulty and recognition (τ_b: *p* = .534). Distractor distributions did not differ across ChatGPT, Google Gemini and NLE (χ^2^(2) = 2.61, *p* = .271, U *p* > .05 for all comparisons). Item difficulty did not differ significantly between AI and NLE items; however, exploratory source-specific analysis showed an overall difference among ChatGPT, Google Gemini, and NLE items (χ²(2) = 6.71, *p* = .035), with a difference between Google Gemini and NLE items (*p* = .028). Student-perceived curricular alignment did not differ significantly between AI and NLE or among ChatGPT, Google Gemini, and NLE.

**Conclusions:**

Students’ recognition did not differ between AI-generated MCQs and NLE MCQs. Easier MCQs are generally perceived as more aligned with the curriculum. AI-MCQs may provide a feasible approach to item drafting within a structured human-review process.

**Supplementary Information:**

The online version contains supplementary material available at https://doi.org/10.1186/s12909-026-10410-8.

## Background

### When “Correct” is not enough

In Alan Turing’s “imitation game”, artificial intelligence (AI) is considered successful when it is indistinguishable from its human counterpart, asking the question, “Can machines think?” [1, 2]. Today, a more detailed follow-up question may arise in medical education: Can students distinguish between questions written by AI and questions phrased by experts, and what does it mean if they cannot? Much of the emerging literature focuses on large language models’ (LLMs) ability to create plausible multiple-choice questions (MCQs) and compare their psychometric properties, looking predominantly at the technical workability of LLMs in assessment contexts [3, 4]. Yet, this study goes beyond the mere feasibility of AI; it concerns the educational value and assessment quality, particularly with regard to constructive alignment and the validity of assessment decisions, in order to paint a more comprehensive image of current AI-based MCQ assessment practice [5, 6]. Exam questions are of practical relevance to medical educators. Creating individual high-quality MCQs is resource-intensive. It is estimated that establishing a single high-quality question can take up to 24 h and cost between USD 1,500 and USD 2,500, with creation, review and quality assurance taken into account [7, 8]. It seems only logical that this has fueled interest in reducing the time required by educators without compromising the quality of assessment [9]. Including AI-assisted exam question generation may be an important step. Insights from stakeholder-oriented implementation work indicate that pedagogical validity and the technical feasibility of generative AI questions must go hand in hand [10]. In particular, their suitability for the curriculum’s learning objectives should be emphasized.

### Revisiting constructive alignment in the age of generative AI

From a constructive standpoint, the quality of assessment depends on the coherence between learning activities (LA), and the assessment [5]. Generative AI may alter how assessment tasks relate to self-controlled activities. Personalized and adapted LA, including feedback, can now be created quickly and therefore become a more common asset in the curricular toolbox [11]. Within these AI-mediated learning environments, curricular alignment may become a more continuous and iterative process across different LA, rather than remaining a fixed construct, thereby contributing to a more diverse curricular playing field [12, 13].

Evaluating LA can be done by those who are being assessed: the learners. Students are important stakeholders when it comes to LA and assessment. They are the ones most affected by assessment decisions. Their understanding of both assessment and their own learning is shaped by those decisions [14]. This perspective is relevant in the evolving AI-mediated learning landscape, where students’ self-regulated learning may be shaped by assessment requirements as well as LLM-based learning resources [15, 16]. Students’ perceptions that summative feedback might be misaligned with their perception of the LA have been associated with negative emotions and may potentially reduce further engagement [17]. When suggesting a change to current practices of MCQ design, it is therefore important that such questions show comparable item difficulty and distractor quality to National Licensing Exam (NLE) MCQs [18, 19]. It is also important to ensure that MCQs are well aligned with the curriculum in order to avoid underrepresentation of curricular content and potential student frustration, as described above [5, 17].

Notwithstanding the rising number of studies on AI-generated learning content and exam questions, there has been limited research into how recognizable such items are to learners [3, 20, 21]. Prior work shows the capability of AI to generate MCQs of plausible quality, as well as perform well on high-stakes examination-style questions [18, 22].

This study examines (1) whether medical students can distinguish between AI-generated and NLE questions created by humans, (2) whether recognition is associated with item difficulty and (3) how students perceive the curricular alignment of the items.

## Methods

An exploratory cross-sectional study was conducted during the Family Medicine (FM) end-of-year exam in summer semester 2024. Eligible participants were regularly enrolled year five students who had successfully completed the FM curriculum at Saarland University. The exclusion criterion was failure to provide informed consent to participate in the study. Participants received a tablet-based exam consisting of 30 AI-generated questions in addition to 30 NLE questions. All NLE were provided by the NLE board and carefully selected based on constructive alignment with the learning objectives of the curriculum. Students’ performance in the exam determined the final grade for the course. Participants were asked immediately after each single-item MCQ or evolving case-based key-feature item (i.e., an item with linked MCQs focusing on clinical decision-making) to respond to two study questions:

1. Source attribution: whether participants believed the previous MCQ was AI-generated or an NLE question
2. Student-perceived alignment: whether the previous MCQ aligned with the curriculum and its learning objectives

Source attribution accuracy was defined as the proportion of responses in which the student could correctly classify an item as AI or NLE. Student-perceived alignment with the course curriculum was dichotomized.

AI questions were generated using OpenAI ChatGPT-4o and Google Gemini 1.5 Pro. Both AIs received the full blended learning course curriculum, and all the provided learning materials and online learning activities (i.e. online commentary, lectures, learning objectives, podcasts, online study material etc.), as well as additional web links for self-study (i.e., external references) [23]. This information was provided in Portable Document Format (PDF). LLMs were instructed to generate five MCQs using the three main topics of the FM blended learning course curriculum (abdominal pain, febrile infection, back pain). The first set of questions was reviewed by the author (PV) and refined using an additional prompt to query higher competency levels (decision-making, clinical reasoning). A subset of questions was selected for the generation of follow-up key-feature items, with the items selected using a random number generator. The sequential prompting workflow is illustrated in Supplementary Fig. 1 (S1).

**Fig. 1:**
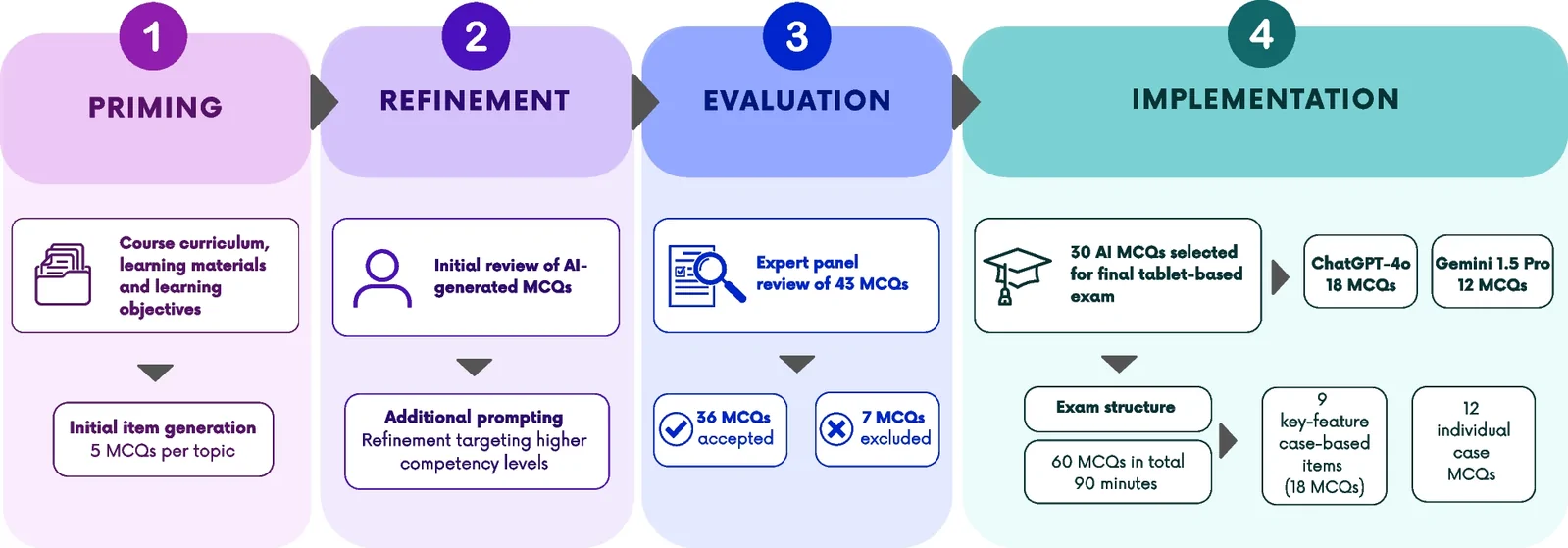
Process from starting MCQ generation to implementing MCQs into the final exam

The LLMs generated 44 MCQs out of which 43 were submitted to a panel of experts (consisting of SJ, SVW, JJ and FD) for review. A total of 36 MCQs (81.8% of the initially generated questions) were accepted for inclusion in the 2024 FM examination, of which 30 questions were used in the written examination (68.2% of the initially generated questions were used in the exam). These 30 questions consisted of 18 questions from ChatGPT and 12 questions from Google Gemini (examples provided in Supplementary Fig. 2 (S2)). A total of 9 questions were asked with a follow-up key-feature question (18 questions in total) and 12 questions were individual case-based questions. All AI MCQ items underwent a validation process by an expert panel, consisting of teaching coordinators, medical education experts and lecturers, as presented in Fig. 1*.* Retained AI-generated items underwent limited expert refinement, including adaptation to the German primary care and guideline context, clarification of clinical details, and minor wording or distractor adjustments. Items requiring substantial content or structural revision were excluded rather than extensively rewritten for inclusion (example provided in Supplementary Fig. 3 (S3)). After the expert panel review, seven MCQs had been eliminated from the question pool and the remaining MCQs were considered suitable for use in the exam.

The IT platform UCAN was used for item management, which automatically randomized the order of exam questions for each student. The final FM exam involved 60 questions and was conducted with a time limit of 90 min in total, thereby simulating a high-stakes examination setting.

Key-feature items consisted of an evolving clinical vignette with linked follow-up MCQs always from the same origin (either AI or NLE) reflecting the case-based structure used in the German NLE [24]. Each key-feature case (stem question and follow-ups) was treated as one clustered item for source recognition and student-perceived curricular alignment. Individual case MCQs were treated as stand-alone items.

The primary analysis was predetermined as source attribution accuracy (AI vs. NLE). Pre-specified secondary analyses examined the association between origin-recognition and item difficulty, as well as student-perceived curricular alignment between AI-generated and NLE items. Additional analyses (distractor distribution and subgroup comparisons) were performed post-hoc and were reported as exploratory. No general adjustment for multiple comparisons was applied to the item-level analyses. Pairwise comparisons in the three-source mixed-effects models were Holm-adjusted (pHolm).

Normality assumption was examined using the Shapiro–Wilk test. Homogeneity of variances was assessed using Levene’s test. In case of non-homogeneous variances, Welch’s t-test (t) was used. Welch’s t-test (t) was performed to compare recognition between groups. Associations between recognition and item difficulty were tested using Kendall’s tau-b (τ_b). Comparisons of student-perceived curricular alignment and item difficulty between two groups (AI vs NLE) were assessed using the Mann–Whitney U test (U), normality was assessed as described above. Mean correct source recognition across 39 items was additionally compared with the 50% chance level using a one-sample t-test (t). Complementary logistic mixed-effects models with random intercepts for student and item were performed at the individual-response level. An intercept-only model assessed definite source recognition against chance (estimated probability with 95% CI, z, p), while source was included as a fixed effect to compare AI and NLE items for recognition and student-perceived curricular alignment (OR, 95% CI). Exploratory post-hoc analysis included:

One-way Fisher's analysis of variance (ANOVA; comparison of AI model recognition between ChatGPT, Google Gemini and NLE), Kruskal–Wallis test (H, reported as χ^2^; comparison of distractor distribution, item difficulty and student-perceived curricular alignment between ChatGPT, Gemini and NLE), Mann–Whitney U test (U; comparisons of distractor distribution), Spearman’s rho (ρ; correlation of item difficulty and student-perceived curricular alignment), chi-square test (χ^2^; distribution of categorized difficulty), source-specific mixed-effects models comparing ChatGPT, Google Gemini and NLE (Omnibus reported as χ^2^).

Item difficulty was quantified by item difficulty index, which is defined as the proportion of students who answered a question correctly (range 0–1; lower values indicate a higher degree of difficulty). In addition, the distribution of answer options was examined to characterize response patterns. Distractor distribution was quantified using a defined index based on incorrect responses (Index: *DD* = *(Pmax-Pmin)/(1-Pcorrect*)). Distractor analysis was used for item quality control [19]. Quantitative data were analyzed using Jamovi 2.3.28.0. Ethics approval was obtained by the Saarland Chamber of Physician's Ethics Board on 24th June 2024, Germany (Bu 234/20).

## Results

### Study population

A total of 119 year five medical students enrolled in the FM course in summer semester 2024 participated. All enrolled students participated, no students were excluded. The analysis included 39 items, consisting of 60 MCQs. 21 AI items included 12 individual case MCQs (6 from ChatGPT 4o, 6 from Google Gemini 1.5 Pro) and 9 clustered key feature items (6 from ChatGPT 4o, 3 from Google Gemini 1.5 Pro). The remaining 18 were NLE items, involving 13 individual case MCQs and 5 clustered key feature items.

### Pre-specified analyses

#### Students’ recognition did not differ between AI and NLE

Descriptive analysis showed mean values for correct recognition were M = 69.6 (SD 15.7) for AI items (*n* = 21) and M = 77.5 (SD 22.7) for NLE items (*n* = 18). Mean values for incorrect recognition were M = 49.4 (SD 15.7) for AI and M = 41.8 (SD 22.8) for NLE.

**Fig. 2:**
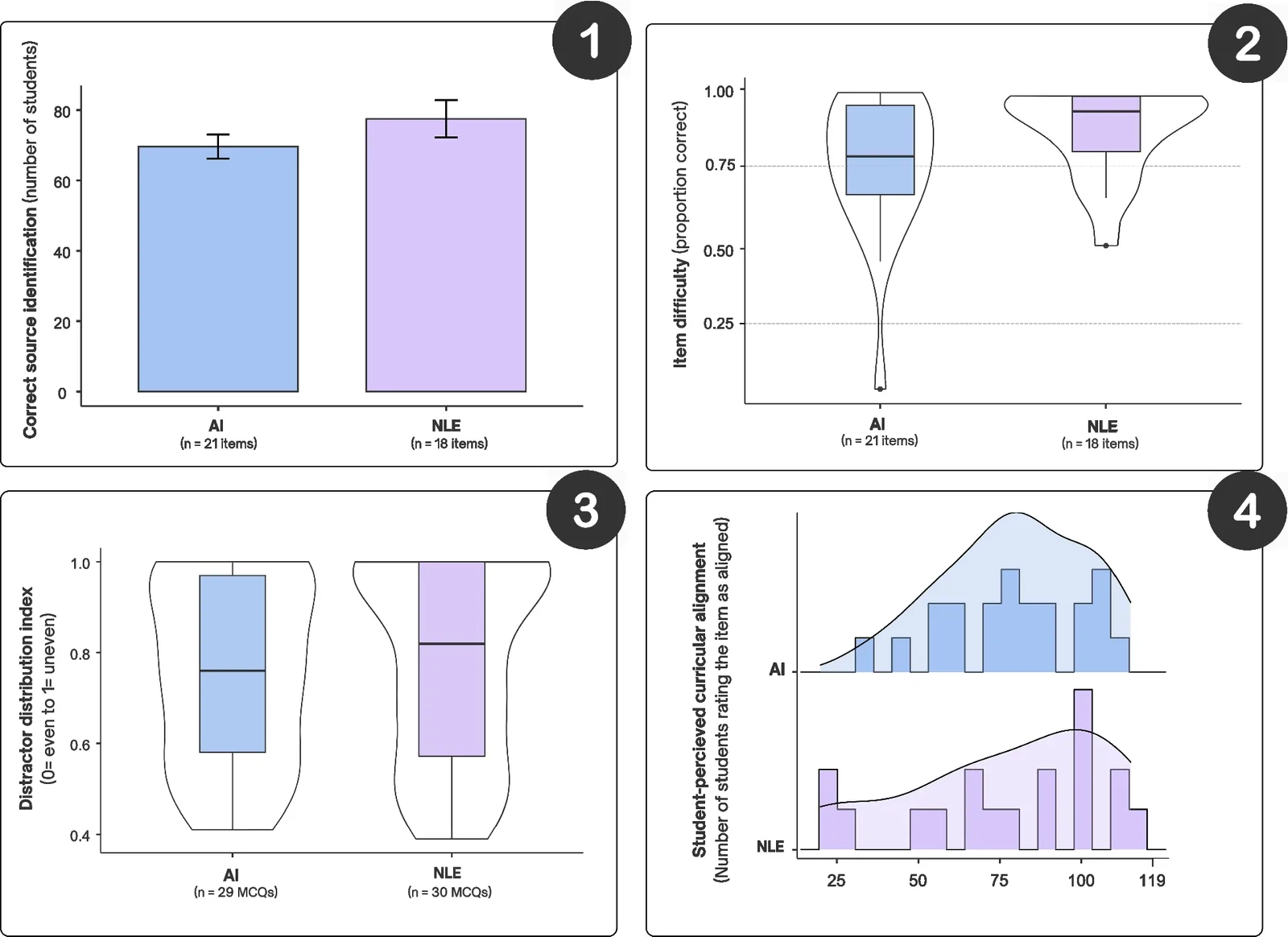
Summary of key results: (1) Correct source recognition, expressed as the number of students correctly identifying the source of each attribution unit. Linked MCQs belonging to the same key-feature case were treated as one item. Error bars indicate 95% CIs. (2) Item difficulty, expressed as the proportion of students answering each item correctly. (3) Distractor distribution index, ranging from 0 (even distribution) to 1 (uneven distribution). (4) Student-perceived curricular alignment, expressed as the number of students rating each item as aligned with the curriculum (AI: *n* = 21, NLE *n* = 18)

Students’ ability to correctly identify a question’s source showed no significant difference between generative AI and NLE MCQs as presented in Fig. 2. Differences were not significant for correct recognition (t(29.6) = −1.24, *p* = 0.225) nor for incorrect recognition (t(29.6) = 1.18, *p* = 0.246). This finding was supported by the complementary mixed-effects analysis, which detected no difference in correct source recognition between AI and NLE items (OR = 1.43, 95% CI 0.88–2.34, *p* = 0.147).

Across the 39 items included in the recognition analyses, mean correct source recognition was 63.9% (95% CI 58.6–69.2%) and was significantly above a 50% chance level (t(38) = 5.30, *p* < 0.001). The complementary mixed-effects analysis of definite individual source attribution estimated a probability of correct recognition of 66.1% (95% CI 60.0–71.7%; z = 5.01, *p* < 0.001).

#### Recognition was not associated with item difficulty

Median item difficulty was Mdn = 0.760 (IQR 0.280) for AI MCQs and Mdn = 0.930 (IQR 0.172) for NLE items as presented in Fig. 2. Item difficulty did not differ significantly between AI-generated questions and NLE items (U = 136, *p* = 0.134, *r* = 0.283). No significant correlation between correct recognition and item difficulty was found (τ_b = 0.071, *p* = 0.534).

#### Student-perceived curricular alignment did not differ between AI and NLE MCQs

Overall, 66% of students’ ratings indicated that the respective item was aligned with the course curriculum, while 34% indicated that it was not aligned. Median alignment scores were similar for AI MCQs and NLE MCQs (AI MCQs: Mdn = 81.0, IQR 34.0, NLE MCQs: Mdn = 84.5, IQR 41.0). In the binary comparison of generative AI vs. NLE, no significant differences were observed (“aligned” U = 188, *p* = 0.978, *r* = 0.008; “not aligned” U = 187, *p* = 0.966, *r* ≈ −0.011). This finding was supported by the complementary mixed-effects analysis, which showed no significant difference in the probability of an item being perceived as aligned between AI and NLE items (OR = 1.04, 95% CI 0.385–2.81, *p* = 0.938).

### Post-hoc exploratory analyses

#### Recognition did not differ by AI model

Descriptive data provided the following mean values for correct recognition: M = 68.6 (SD 14.3) for ChatGPT (*n* = 12), M = 71.0 (SD 18.3) for Google Gemini (*n* = 9), and M = 77.5 (SD 22.7) for NLE (*n* = 18).

In the exploratory analysis, recognition between two different LLMs and the NLE MCQs did not differ (ANOVA; *p* = 0.443 for correct recognition;* p* = 0.474 for incorrect recognition). The source-specific mixed-effects model likewise showed no overall effect of source on correct recognition (χ^2^(2) = 2.17, *p* = 0.338). Holm-adjusted pairwise comparisons showed no significant differences between sources (ChatGPT vs. NLE: OR = 1.49, 95% CI 0.65–2.33, pHolm = 0.499; Gemini vs. NLE: OR = 1.36, 95% CI 0.52–2.21, pHolm = 0.665; ChatGPT vs. Gemini: OR = 0.91, 95% CI 0.31–1.52, pHolm = 0.792).

#### Categorized difficulty did not differ between AI and NLE items

Across two different categorizations of difficulty, no significant difference in distribution was detected between AI and NLE items (10% χ^2^ (8) = 7.63, *p* = 0.470, 20% χ^2^ (4) = 4.48, *p* = 0.345).

#### Item difficulty differed across sources

The descriptive data on item difficulty across the sources provided the following median scores: Mdn = 0.890 (IQR 0.212) for ChatGPT (*n* = 12), Mdn = 0.730 (IQR 0.180) for Google Gemini (*n* = 9) and Mdn = 0.930 (IQR 0.172) for NLE (*n* = 18). Item difficulty differed significantly across the three sources (χ^2^(2) = 6.71, *p* = 0.035, ε^2^ = 0.177). Pairwise comparisons indicated a significant difference between Google Gemini and NLE (*p *= 0.028), whereas no significant difference was observed between ChatGPT and NLE (*p* = 0.984), as well as ChatGPT and Google Gemini (*p* = 0.123).

#### Item difficulty was strongly associated with student-perceived alignment

Item difficulty and perceived alignment showed a strong association; easier items were seen as better aligned (*ρ* = 0.762, *p* < 0.001) and items with higher difficulty were perceived as “not aligned” (*ρ* = —0.762, *p* < 0.001).

#### Source-specific student-perceived curricular alignment did not differ

Median alignment scores were comparable across ChatGPT, Google Gemini and NLE (“aligned” ChatGPT: Mdn = 90.5, IQR 26.0, Google Gemini: Mdn = 75, IQR 21.0, NLE: Mdn = 84.5, IQR 41.0). Additionally, in a source-specific analysis no significant difference was observed across the three sources (“aligned” χ^2^ (2) = 4.03, *p* = 0.134, ε^2^ = 0.11; “not aligned” χ^2^ (2) = 3.99, *p* = 0.136, ε^2^ = 0.11). This finding was supported by the complementary mixed-effects analysis, which likewise detected no overall effect of source on student-perceived curricular alignment (χ^2^ (2) = 3.88, *p* = 0.144; ChatGPT vs. NLE: OR = 1.69, 95% CI 0.56–5.07; Gemini vs. NLE: OR = 0.46, 95% CI 0.14–1.53). Holm-adjusted pairwise comparisons likewise showed no significant difference between ChatGPT, Google Gemini and NLE items (all pHolm ≥ 0.147).

#### Distractor distribution did not differ across MCQ origin

Distractor distribution showed no difference across MCQ origins as presented in Fig. 2. Median scores of distractor distribution were 0.840 (IQR 0.400; *n* = 17) for ChatGPT, 0.725 (IQR 0.245, *n* = 12) for Google Gemini, and 0.820 (IQR 0.427; *n* = 30) for NLE.

No significant differences in a three-way comparison occurred (χ^2^ (2) = 2.61, *p* = 0.271; ε^2^ = 0.045). Combined AI comparison with NLE distractor distribution did not differ (AI: Mdn = 0.760, IQR 0.390; *n* = 29 vs. NLE Mdn = 0.820, IQR 0.427; *n* = 30, U = 394, *p* = 0.529, *r* = 0.095).

## Discussion

### Can students distinguish AI?

 If Turing’s “imitation game” is taken as a framework for assessing whether AI output can be distinguished from human-generated output, our findings provide a nuanced result: students identified item source at a rate above chance, but recognition remained imperfect and did not differ between AI-generated and NLE items. When considering other measured item characteristics, no differences were detected between AI and NLE items in the measured indicators, particularly when looking at item difficulty, student-perceived curricular alignment, and distractor distribution. Beyond source attribution, the findings also suggest that students’ judgment of curricular alignment may be shaped by item difficulty. Easier items were more often perceived as aligned, whereas more difficult items were rated as not aligned. Another interesting aspect is that there seems to be a discrepancy between the students’ self-assessment after the exam (many students reported that they could “recognize AI questions very easily”) and their actual performance in recognizing AI. This highlights the care needed when implementing AI in curricula, as non-recognition could lead to uncritical use on both sides [25, 26].

### Practical significance: shift in workload for educators

The reality appears to be more diverse. In the literature, item writing is found to be time-consuming and effort intensive [9, 27]. The use of LLMs in the drafting stages has also been well described [9, 27], suggesting resources may be freed up as educators shift from MCQ drafting to reviewing.

This study used an expert-panel workflow that supports a procedure where AI-generated MCQs serve as template, while humans remain responsible for quality control, i.e. in terms of content accuracy or distractor plausibility. The necessity of human oversight is shown in this study by the fact that 18.2% of the initially generated MCQs had to be eliminated. This is consistent with previous reports, where human evaluation is consistently mentioned as an important component due to AI errors [28, 29].

Besides AI-generated errors, a visible bias underestimating FM competency was also observed during the human evaluation process. In this study e.g. distractors of generative AI MCQs suggested referrals to other specialists for abdominal ultrasound, which is a common FM task in daily practice. Bias can emerge in AI output mostly via the content of training-data such as representation gaps [30, 31]. If AI generated content is indistinguishable, it can give a false sense of neutrality and fairness. The practical consequence is straightforward: integrating LLMs into item generation should involve a thoughtful evaluation, also with the concept of structural biases in mind.

### From summative assessment to a broader medical education

These implications are particularly relevant for assessment and feedback culture in countries such as Germany, where NLE and medical education are heavily focused on MCQs [32]. In systems that strongly rely on summative assessments, improvements in item development could have a significant impact. The potential value of AI may extend beyond summative assessments. For example, AI-supported item generation could be used in a formative context to create practice questions while also providing explanations and feedback for distractors [16, 28, 31]. More structured, repeated formative LA may also be created [33]. The generation of practice questions with accompanying feedback may be supported by AI question drafting [34, 35]. Potential resources freed up by AI capabilities could therefore be redirected into broader feedback and learning formats.

### Strengths and limitations

This study can add to the literature in three ways:

1. Its integration into a regular exam setting complements previous studies in a realistic way.
2. Using two different LLMs reduced dependence on the characteristics of a single model and broadened the comparison.
3. The inclusion of key-feature questions extends assessment formats beyond conventional MCQs by capturing items that focus more specifically on clinical reasoning and decision-making.

This study addresses the distinguishability of AI-generated items in authentic exam situations. Conducting the study at a single institution, in one subject and one cohort limits transferability to other faculties, subjects, or groups of students. This also applies to the observed variation in item difficulty across item sources. Although an overall difference across item source was found, pairwise differences were limited and driven by the contrast between Google Gemini and NLE items. This finding should therefore be interpreted with caution, as the limited number of items per source increases the influence of individual outliers (one Google Gemini item showed a particularly low difficulty index). Additionally, the prior exposure of participants to NLE items (e.g. via commercial learning platforms) may have influenced students’ source attribution without this effect being fully controllable. These results show a strong association between higher item-difficulty index values (i.e., easier items) and greater student-perceived curricular alignment. This limits the interpretation and raises the possibility that subgroups (e.g. higher vs. lower performers) differ in their observed curricular alignment.

Future studies should examine how AI assessments can influence students’ learning behavior, what effects the observed specialty bias in curricular AI implementation can have, and how review processes can be structured and ensure reproducibility.

## Conclusions

In realistic high-stakes exam settings, students identified item source at a rate significantly above chance, although recognition accuracy remained moderate and did not differ between AI-generated and NLE items. Measured quality indicators for item control (difficulty index, student-perceived curricular alignment, distractor distribution) did not differ between AI and NLE items, suggesting potential usability in an exam setting. The findings from this study suggest maintaining human oversight to minimize AI mistakes and avoid a possible specialty bias. AI-MCQ drafting may provide opportunities to redirect resources into broader curricular developments and improvements by potentially freeing up educational resources while maintaining structured expert review of AI-generated MCQs. AI-assisted drafting may also provide more structured repetitive testing LA with formative feedback, as MCQ and feedback creation can go hand in hand with AI-assisted drafting.

## Supplementary Information

Supplementary Material 1: Supplementary Fig. 1 (S1). Illustration of the prompting workflow for AI-MCQ generation. Prompts were entered sequentially. Each step builds on the previous output and refines the request. Example prompts were abbreviated and translated into English.

Supplementary Material 2: Supplementary Fig. 2 (S2). AI-generated MCQ examples, reviewed and edited, used in the final exam. Example 1 originated from ChatGPT-4o, Example 2 originated from Gemini 1.5 Pro.

Supplementary Material 3: Supplementary Fig. 3 (S3). Example of an AI-generated MCQ before and after expert panel review. The final version was used in the examination. Modified sections are highlighted in blue.

## Data Availability

The datasets generated and/or analysed during the current study are not publicly available due to restricted access. The data are available from the corresponding author upon reasonable request and subject to applicable institutional, ethical, and data protection requirements.
