# Supplementary material for: Can students identify AI? – A cross-sectional quantitative study about AI recognition in tablet-based MCQ assessment among fifth-year undergraduate medical students at Saarland University, Germany
Source: BMC Med Educ. 2026 Sep 24;26:1519. doi: 10.1186/s12909-026-10410-8 (PMC13602609; doi:10.1186/s12909-026-10410-8)
Supplement: Supplementary file 1 [file 12909_2026_10410_MOESM1_ESM.pdf]

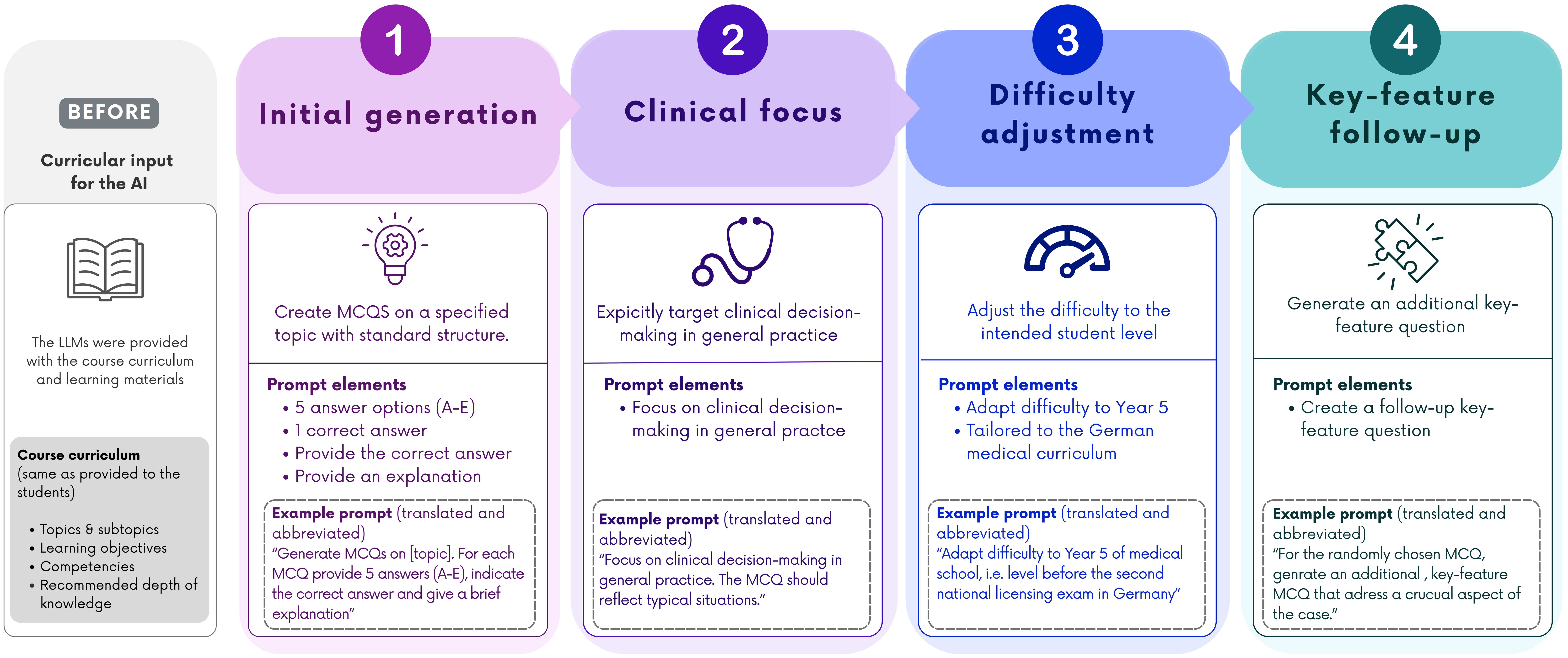

Supplementary Figure 1 (S1)

Illustration of the prompting workflow for AI-MCQ generation. Prompts were entered sequentially. Each step builds on the previous output and refines the request. Example prompts were abbreviated and translated into English.
