# Supplementary material for: Can students identify AI? – A cross-sectional quantitative study about AI recognition in tablet-based MCQ assessment among fifth-year undergraduate medical students at Saarland University, Germany
Source: BMC Med Educ. 2026 Sep 24;26:1519. doi: 10.1186/s12909-026-10410-8 (PMC13602609; doi:10.1186/s12909-026-10410-8)
Supplement: Supplementary file 2 [file 12909_2026_10410_MOESM2_ESM.pdf]

Examples of AI-generated MCQs used in the exam

ChatGPT-4o

Clinical Case: A 52-year-old man presents with a sudden high fever of up to 40°C, severe body aches, headache, and significant weakness. He reports that he has not recently been vaccinated against the flu or COVID-19. He lives alone and works in an office. The physical examination reveals a generally poor overall condition, but no specific pathological findings.

Question: Which of the following treatment options is most appropriate?

- (A) Prescribing broad-spectrum antibiotics to prevent a possible bacterial superinfection
- (B) Symptomatic treatment with acetaminophen to reduce fever and pain, along with adequate fluid intake
- (C) Immediate administration of a flu vaccine to prevent future infection
- (D) Ordering a magnetic resonance imaging (MRI) scan of the head to rule out other causes of the headache
- (E) Performing a throat swab to rule out a streptococcal infection

Gemini 1.5 Pro

Clinical Case: 45-year-old patient with a history of hyperlipidemia and obesity presents with acute, colicky pain in the right upper abdomen. The pain began after a heavy dinner and radiates to the right shoulder. The patient reports nausea, vomiting, and a positive Murphy's sign. He also reports lighter-colored stools and darker urine over the past few days. On physical examination, you palpate a distended, tender mass beneath the right costal margin.

Question: Which of the following measures is most likely indicated in primary care practice and is consistent with current guidelines?

- (A) Hospital admission for diagnostic ERCP and treatment
- (B) Performing an abdominal ultrasound and, if cholecystolithiasis is confirmed by ultrasound, initiating a calculated oral antibiotic regimen with ciprofloxacin and metronidazole
- (C) Performing an abdominal ultrasound and, if cholecystolithiasis is confirmed by ultrasound, immediate referral to the surgical outpatient clinic for cholecystectomy
- (D) Symptomatic therapy with metamizole and butylscopolamine and close monitoring of symptoms and laboratory values
- (E) Performing an abdominal ultrasound, determining inflammatory markers and liver function tests in the laboratory, and hospital admission

Supplementary Figure 2 (S2). AI-generated MCQ examples, reviewed and edited, used in the final exam. Example 1 originated from ChatGPT-4o, Example 2 originated from Gemini 1.5 Pro
