# Supplementary material for: Can students identify AI? – A cross-sectional quantitative study about AI recognition in tablet-based MCQ assessment among fifth-year undergraduate medical students at Saarland University, Germany
Source: BMC Med Educ. 2026 Sep 24;26:1519. doi: 10.1186/s12909-026-10410-8 (PMC13602609; doi:10.1186/s12909-026-10410-8)
Supplement: Supplementary file 3 [file 12909_2026_10410_MOESM3_ESM.pdf]

AI-generated question before and after expert panel review

BEFORE

Clinical Case: Lisa, 17 years old, comes to your office with a sore throat that has lasted for 9 days, a fever, and severe fatigue. Her tonsils are severely reddened and coated, and she has painful, swollen lymph nodes in her neck. A rapid strep test was negative.

Question: According to current guidelines, which of the following diagnostic measures is the best course of action to confirm the suspected diagnosis of mononucleosis in Lisa?

A. Performing a rapid Epstein-Barr virus (EBV) antibody test

B. Performing a differential blood count and detection of atypical lymphocytes

C. Performing an abdominal ultrasound

D. Ordering a computed tomography (CT) scan of the neck

E. Referring her for a tonsillectomy

AFTER

Clinical Case: Lisa, 17 years old, comes to your office with a sore throat that has lasted for 9 days, a fever, and severe fatigue. Her tonsils are severely reddened and coated, and she has painful, swollen lymph nodes in her neck. A rapid strep test was negative.

Question: According to current guidelines, which of the following diagnostic measures is the best course of action to confirm the suspected diagnosis of mononucleosis in Lisa?

A. Performing a rapid nasopharyngeal Epstein-Barr virus (EBV) antibody test

B. Performing a differential blood count and EBV serology

C. Performing an abdominal ultrasound

D. Ordering a computed tomography (CT) scan of the neck

E. Referring her for a tonsillectomy

Supplementary Figure 3 (S3). Example of an AI-generated MCQ before and after expert panel review. The final version was used in the examination. Modified sections are highlighted in blue.
